# Simultaneous determination of five essential amino acids in plasma of Hyperlipidemic subjects by UPLC-MS/MS

**DOI:** 10.1186/s12944-020-01216-8

**Published:** 2020-03-23

**Authors:** Zhibin Chen, Feiyan Lin, Xuemei Ye, Yuqian Dong, Lufeng Hu, Aifang Huang

**Affiliations:** 1 Department of Nephrology, The Affiliated Yueqing Hospital of Wenzhou Medical University, Yueqing, Zhejiang, China; 2 School of Medicine, Zhejiang University, Zhejiang, Hangzhou China; 3 Department of Pharmacy, The First Affiliated Hospital of Wenzhou Medical University, Nanbaixiang Street, Ouhai District, Wenzhou, 325000 China; 4 School of Pharmacy, Wenzhou Medical University, Wenzhou, 325000 China

**Keywords:** Amino acid, Human, UPLC-MS/MS, Hyperlipemia, Plasma

## Abstract

**Background:**

Millions of adults have been reported with hyperlipemia in the world. It is still unclear whether the plasma level of essential amino acids (AAs) will be influenced by the hyperlipemia. This study was aimed to investigate the AAs levels and the underlying metabolic relationship in hyperlipidemic subjects.

**Methods:**

An ultra-high performance liquid chromatography-tandem mass spectrometric (UPLC-MS/MS) method was developed for the determination of phenylalanine (Phe), valine (Val), histidine (His), tryptophan (Trp), and methionine (Met). Plasma samples (100 μL) were precipitated by acetonitrile (300 μL) and analyzed on a BEH C18 (2.1 mm × 100 mm, 1.7 μm) column at 40 °C by gradient elution. The mobile phase composed of 0.1% formic acid and acetonitrile was used with flow rate at 0.2–0.4 ml/0-3 min. Five AAs were determined at positive electrospray ionization (ESI+) at m/z 118.1/72.1 (Val), 150.12/104.02(Met), 156.06/110.05(His), 166.1/120.1(Phe), and 205.2/188.02 (Trp). A total of 75 healthy subjects and 83 hyperlipidemic subjects, who had blood routine test and plasma lipid test were determined by developed UPLC-MS/MS.

**Results:**

It was shown that there was good linearity for Val, Met, His, Phe, and Trp within 1–100 μg/mL. The relative standard deviations of precision and accuracy were all within 15%. The level of Val, Phe, Trp, His, and Met were 35.34 ± 15.64, 22.72 ± 9.13, 17.23 ± 4.94, 16.78 ± 13.64, and 6.24 ± 1.97 μg/mL in healthy subjects, while they were 38.04 ± 16.70, 22.41 ± 8.45, 15.62 ± 5.77, 18.35 ± 14.49, and 6.21 ± 1.97 μg/mL in hyperlipidemic subjects respectively. The Spearman’s correlations analysis showed that there were high correlations between Val, Phe, Trp, His, Met and triglyceride in healthy subjects. While, those correlations decreased in hyperlipemia cases.

**Conclusion:**

A convenient and sensitive method for simultaneous determination of Val, Phe, Trp, His, and Met in human plasma was developed. There was a high correlation between Val, Phe, Trp, His, Met and triglyceride. Hyperlipemia influences the metabolic balance of His, Phe, Trp, Met and Val.

## Introduction

Amino acid exists in two stereoisomeric forms, L forms and D forms, most of which are L-isomers. The amounts of D-AAs are dramatically low in human body [1]. So far, there are about 500 α-amino acids were discovered in nature. However, only 20 of them are existed in human being [2]. Among them, 9 essential AAs cannot synthesized in human body, which are phenylalanine (Phe), valine (Val), histidine (His), tryptophan (Trp), methionine (Met), lysine, leucine, isoleucine, and threonine [3]. Essential AAs are the most important essential substances in organisms, which serve as the precursors for synthesis of proteins, various enzymes, hormones, and body fluids, et c[4] Moreover, the essential AAs act as the key regulators of gene expression, and participate in various physiological and pathological processes, such as growth, digestion, and maintenance of the body’s immune system [5–7]. Therefore, the study of essential AAs has always been widely concerned in biomedical research.

So far, various analytical methods for quantification of AAs have been reported, such as.

gas chromatography with mass spectrometry (GC-MS) detection [8–10], capillary electrophoresis with contactless conductivity detection [11], UV-absorption detection [12], laser-induced fluorescence detection [13], high performance liquid chromatography (HPLC) with ultraviolet [14] and fluorescence detection [15–17]. Among them, many methods include a derivatization step, such as ethyl chloroformate derivatization [8], methyl chloroformate derivatization [10], trimethylsilyl-trifluoroacyl derivatization [9], since AAs have small molecular weight and strong polarity. However, derivatization is complex and time consuming, and reduces the degree of ionization in the positive ion mode [18]. Compared with GC-MS and HPLC, liquid chromatography tandem with mass spectrometry detection (LC-MS) had higher sensitivity and stability. So far, there several LC-MS methods have been reported [19–23]. However, some methods were used in food [21, 22], some methods had high limits of detection or need derivatization to improve the chromatographic separation [19, 20].

Hyperlipemia is a primary and major risk factor for the atherosclerotic cardiovascular diseases. Millions of adults have been reported with elevated total cholesterol (TC) or triglyceride (TG) levels in the world [24, 25]. Although numerous researches have been carried out to study hyperlipemia, it is unclear whether the plasma levels of essential AAs are affected. Therefore, the purpose of this study is to establish a rapid, convenient, and sensitive method for the determination of essential AAs in human plasma without derivatization. Based on the developed UPLC-MS/MS method, the essential AAs levels in healthy subjects and in hyperlipidemic patients were determined and analyzed.

## Methods

### Reagents

Phe, Val, His, Trp, Met and colchicine (purity > 98%) were bought from Sigma-Aldrich (Lewis, USA) and J&K Scientific LTD (Peking, China). Acetonitrile, methanol, and formic acid were HPLC graded and bought from Merck Company (Darmstadt, Germany). Ultra-pure water (resistance > 18 mΩ) were provided by a Millipore Milli-Q purification system (Bedford, USA).

### Hyperlipidemic and healthy subjects

The blood samples of hyperlipidemic and healthy subjects were collected from the clinical laboratory of the First Affiliated Hospital of Wenzhou Medical University. The study was carried out in accordance with the Declaration of Helsinki, and approved by the ethics committee of the First Affiliated Hospital of Wenzhou Medical University.

All of these subjects received blood routine test (BRT) and lipid test, which were analyzed by the XE-2100 automated hematology analyzer (Sysmex, Japan) and the Beckman AU5800 biochemical measurement (Beckman Coulter, Inc. USA). After BRT and biochemical test, 100 μL of residual plasma was collected and determined by UPLC-MS/MS method.

### UPLC-MS/MS conditions

An ACQUITY UPLC and Xevo TQ-S Micro triple quadrupole mass spectrometer (Waters, USA) was used in this study. Chromatographic separations of His, Met, Trp, Val, Phe and colchicine (internal standard, IS) were separated at a Waters BEH C18 column (2.1 mm × 100 mm, 1.7 μm), which temperature was set at 40 °C. The mobile phase consisted of 0.1% formic acid water (solvent A), which was prepared by added 0.5 mL formic acid into 500 mL water, and acetonitrile (solvent B). The gradient elution of mobile phase began with A: B = 90:10 v/v, which changed as follows: A:B = 80:20 v/v at 1.2 min, A:B = 10: 90 v/v at 2.5 min, A:B = 90:10 v/v at 3.0 min.The flow rate was set at 0.2 mL/min (0–1.2 min), 0.4 mL/min (1.2–2.5 min), 0.2 mL/min (2.5–3.0 min) respectively. The injection volume was 1 μL. His, Met, Trp, Val, Phe and IS were detected in the multiple reaction monitoring (MRM) mode.

### Sample preparation

A total of 100 μL plasma was added into 1.5 mL centrifuge tube. After that, the plasma was precipitated by 300 μL acetonitrile, which contained 0.1 μg/mL of colchicine (IS). The mixture was put under vortex movement for 0.3 min and centrifuged at 15000 rpm for 5 min. Finally, 1 μL supernatant injection was set for UPLC-MS/MS system analysis.

### Calibration curve

The mixed stock solution of His, Met, Trp, Val, and Phe was dissolved with water at 1.00 mg/mL, and diluted to 10-500 μg/mL. The calibration standards were from 1 to 100 μg/mL, which were prepared by spiking 10 μL different concentration of mixed AAs standard solutions into 90 μL of blank plasma. The final concentrations of added AAs in blank plasma were 1, 2.5, 5, 10, 25, 50, 100 μg/mL, which were precipitated by 300 μL acetonitrile as mentioned in sample preparation.

### Method validation

The method validation was evaluated by three quality-control (QC) samples at 10, 40, and 80 μg/mL, which were prepared as samples of calibration curve.

The intra-day precision and accuracy of His, Met, Trp, Val, and Phe was carried out three replications in a day, and the inter-day precision was evaluated by measuring daily and continuously for 3 days. The concentrations of QC samples were calculated by the calibration curves developed. The precision was expressed as the relative standard deviation (RSD), and the accuracy was expressed as the relative error (RE), which was calculated as: RSD = Mean QC/SD × 100% and RE = (Mean QC - QC)/QC × 100%.

The extraction recovery at 10, 40, and 80 μg/mL was evaluated by comparing the peak area to the pure standard solution at the same concentration. The matrix effects were investigated by comparing the peak area of His, Met, Trp, Val and Phe to the extracted sample added with the same concentration of analytes. The stability was evaluated at the room temperature from 2 h to 24 h.

The robustness of UPLC-MS/MS method was studied in term of mobile phase, pH, column temperature and flow rate at three QC sample concentrations. Those parameters were interchanged within the range of 1–10% of the developed conditions, while keeping the other parameters unchanged. The peak areas of 5 AAs were recorded. The ruggedness was determined on two different BEH C18 Columns, centrifuges and by two different analysts to perform the overall analysis.

### Data analysis

All data of BRT, lipids indices and five AAs were expressed as mean and standard deviation. Independent sample t test was used to analyze the difference between hyperlipidemic and healthy subjects. The relationships of 5 AAs were analyzed by bivariate correlation. The statistical analysis was carried out by using the statistical software SPSS 17 (IBM, USA).

## Results

### Method development and linearity

**Fig. 1:**
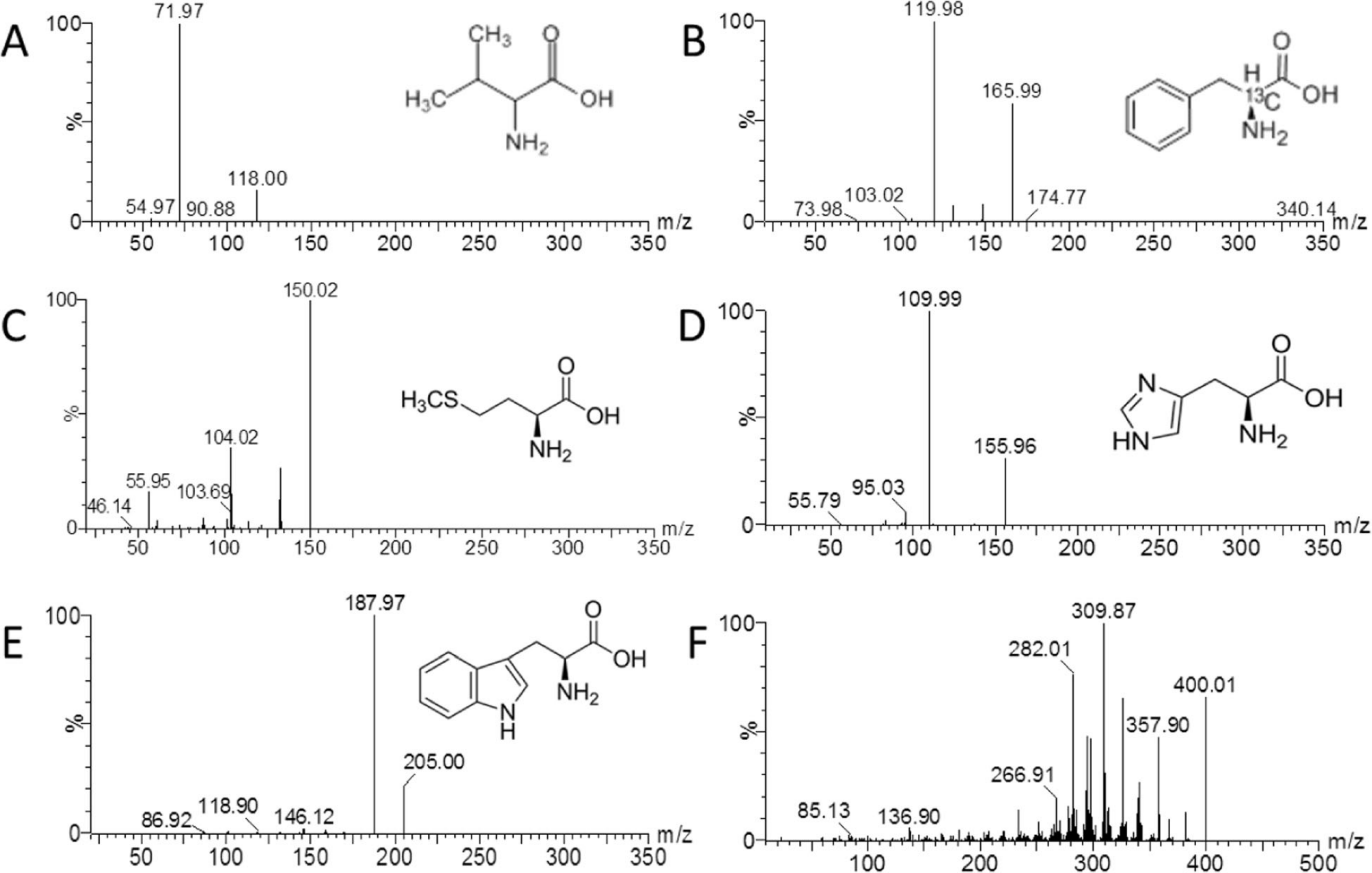
Chemical structures and mass spectra of Val (**a**), Phe (**b)**, Met (**c)**, His (**d)**, (**e**) Trp and IS (**f**)

In this study, Val, Met, His, Phe, and Trp all had characteristic daughter ions in positive model. The optimal collision energy of five AAs was from 10 to 15 eV. If the collision energy was over 20 eV, the MS response will decrease. The developed UPLC-MS/MS parameters and mass spectrum are shown in Table 1 and Fig. 1.

**Table 1:** UPLC-MS/MS data of five essential AA and colchicine (IS)

Compound	ion mode	parent ion (m/z)	daughter ion (m/z)	Cone(V)	Collision
Val	ESI+	118.10	72.10	40	15
Met	ESI+	150.12	104.02	40	10
His	ESI+	156.06	110.05	40	15
Phe	ESI+	166.10	120.10	20	15
Trp	ESI+	205.20	188.02	20	10
IS	ESI+	400.00	358.00	40	20

**Fig. 2:**
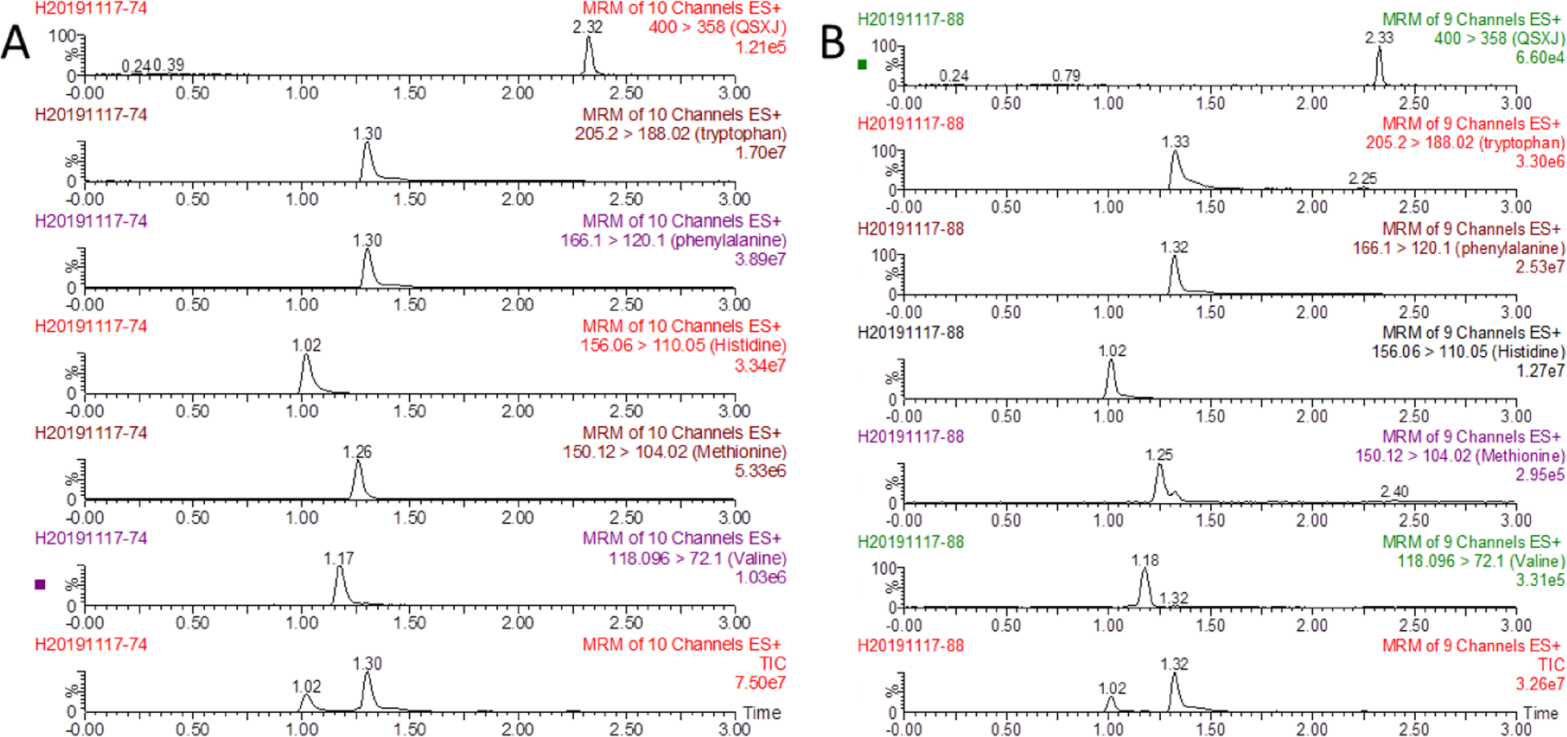
UPLC-MS/MS and total ion chromatogram of His, Met, Trp, Val, Phe and IS in pure amino acids and blood sample

According to the selected UPLC-MS/MS condition, the result showed that there was no interfering endogenous substance observed in the UPLC-MS/MS chromatograms (Fig. 2).

The calibration curve was developed by the linear regression of peak area ratios against the concentrations. Since AAs are endogenous substances, their calibration curves were subtracted with the area of a blank sample. The equation of calibration curves were showed in supplement Table 1. The correlation coefficients (R) of His, Met, Trp, Val, Phe were 0.9954, 0.9984, 0.9980, 0.9985, and 0.9942 over the concentration ranges. The lower limit of quantitation was 1 μg /mL for His, Met, Trp, Val and Phe.

### Method validation

The intra-day and inter-day precision and accuracy of His, Met, Trp, Val, Phe at the three QC levels in plasma are presented in Table 2. The results showed that RSD of intra-day and inter-day precision of 5 AAs were all within 15%. RE of accuracy ranged from − 7.63 to 12.12%.

**Table 2:** intra-day, inter-day precision and accuracy of 5 AA in human plasma (*n* = 4)

AA	QC (μg/mL)	Intra-day			Inter-day			
		Mean ± SD (μg/mL)	Precision (RSD %)	Accuracy (RE %)	Mean ± SD (μg/mL)	Precision (RSD %)		Accuracy (RE %)
his	10	11.21 ± 1.21	10.78	12.12	10.36 ± 1.48	14.27		3.62
	40	43.23 ± 6.41	14.83	8.07	42.04 ± 4.46	10.60		5.09
	80	82.06 ± 8.59	10.47	2.57	81.34 ± 9.20	11.31		1.68
met	10	10.81 ± 1.55	14.35	8.08	10.30 ± 1.44	13.96		3.01
	40	41.48 ± 4.87	11.73	3.71	41.43 ± 5.44	13.13		3.56
	80	82.05 ± 12.29	14.97	2.57	88.00 ± 11.05	12.55		10.00
val	10	11.04 ± 1.44	13.01	10.39	11.02 ± 1.59	14.46		10.22
	40	41.95 ± 1.17	2.79	4.87	45.14 ± 6.72	14.88		12.85
	80	80.61 ± 3.80	4.71	0.76	85.37 ± 10.04	11.76		6.72
trp	10	9.55 ± 1.26	13.19	−4.52	10.07 ± 0.55	5.50		0.69
	40	43.46 ± 6.05	13.93	8.64	41.13 ± 2.32	5.64		2.84
	80	77.89 ± 10.16	13.04	−2.64	74.07 ± 4.40	5.94		−7.42
phe	10	10.42 ± 1.45	13.91	4.19	10.17 ± 1.08	10.58		1.66
	40	36.95 ± 5.12	13.87	−7.63	38.17 ± 5.60	14.67		−4.59
	80	78.77 ± 5.28	6.70	−1.53	78.31 ± 5.20	6.63		−2.12

The extraction recovery, matrix effect and stability of the five essential AAs are presented in supplement Table 2. The mean extraction recoveries of His, Met, Trp, Val, Phe at three different QC levels were 76.48 ± 6.88, 70.10 ± 5.55, 70.96 ± 6.47, 73.40 ± 9.10, 74.46 ± 10.61, 72.49 ± 6.60, which indicated that there was no obvious difference among the extraction recoveries of His, Met, Trp, Val, Phe. The matrix interference ranged from 62.15 to 84.14%, which indicated that the matrix effect was acceptable. The extract stability showed each QC level below 15% deviation from initial concentrations, which demonstrated that they were stable in plasma at room temperature for 24 h.

The results of robustness and ruggedness revealed there were no significant changes observed in the chromatographic peaks of 5 AAs. The peak RSD values of mobile phase, pH, column temperature and flow rate were all < 15% (Supplement Table 3), and the RSD values of ruggedness were all < 15% (Supplement Table 4). The results indicated that there was no significant effect on the determination of 5 AAs when the chromatographic conditions were changed slightly.

### Clinical indices of Hyperlipidemic and healthy subjects

A total of 75 healthy subjects (male 32, female 43) and 83 hyperlipidemic subjects (male 40, female 43), aged between 18 and 60 years, were participated in the study. Their average ages were 37.19 ± 9.66 and 44.77 ± 9.22. The clinical indices of two groups were listed in Table 3. There were no abnormal changes in BRT indices of hyperlipidemic and healthy subjects. Although the levels of RBC and HB had risen slightly in hyperlipidemic group, they were still in the normal range. However, the blood levels of TG, TC, and LDL were all obviously increased (*P* < 0. 001).

**Table 3:** BRT and lipids indices of hyperlipidemic and healthy subjects (mean ± SD)

index	healthy	hyperlipidemic
White blood cell(10*9/L)	5.89 ± 1.38	6.07 ± 1.31
Absolute value of monocyte(10*9/L)	0.42 ± 0.14	0.43 ± 0.15
Red blood cell (10*12/L)	4.67 ± 0.41	4.83 ± 0.42*
Hematokrit (%)	0.42 ± 0.03	0.43 ± 0.05
Percentage of leukomonocyte (%)	0.35 ± 0.08	0.35 ± 0.07
Absolute value of leukomonocyte (10*9/L)	2.04 ± 0.52	2.13 ± 0.54
Mean corpuscular hemoglobin (pg)	69.04 ± 332.13	30.52 ± 1.08
Mean corpuscular hemoglobin concentration (g/L)	341.64 ± 8.64	334.53 ± 48.27
Absolute value of basophilic granulocyte (10*9/L)	0.01 ± 0.01	0.01 ± 0.02
Absolute value of eosinophils (10*9/L)	0.19 ± 0.50	0.16 ± 0.13
Hemoglobin (g/L)	141.80 ± 11.79	147.42 ± 13.28*
Blood platelet (10*9/L)	239.91 ± 47.05	238.14 ± 49.71
Thrombocytocrit (%)	0.26 ± 0.05	0.26 ± 0.05
Percentage of neutrophile granulocyte (%)	8.79 ± 71.41	0.55 ± 0.07
Absolute value of neutrophile granulocyte (10*9/L)	3.22 ± 1.13	3.34 ± 0.95
Red cell volume distribution width (%)	12.72 ± 0.57	12.78 ± 0.54
Total cholesterol (mmol/L)	4.39 ± 0.54	5.80 ± 0.87**
High density lipoprotein (mmol/L)	1.23 ± 0.27	1.23 ± 0.30
Low density lipoprotein (mmol/L)	2.50 ± 0.50	3.38 ± 0.77**
Triglyceride (mmol/L)	1.01 ± 0.31	1.80 ± 1.28**

Note: Compared with healthy subjects: *P < 0.05, **P < 0.001

### Levels of 5 AAs in Hyperlipidemic and healthy subjects

Based on the UPLC-MS/MS method, the levels of 5 essential AAs in the hyperlipidemic subjects and the healthy subjects were determined. The plasma levels of 5 essential AAs were shown in Table 4. It could be found that the mean blood levels of Val, Phe, Trp, His, and Met were different. Among them, the levels of Val and Phe were higher than those of Trp, His and Met. For hyperlipidemic subjects, the blood levels of Val and His increased and the blood level of Trp decreased.

**Table 4:** Plasma level of 5 AAs between hyperlipidemic and healthy subjects (mean ± SD)

Fatty acid	Unit	healthy subjects	hyperlipemia subjects
Val	μg/mL	35.34 ± 15.64	38.04 ± 16.70
Phe	μg/mL	22.72 ± 9.13	22.41 ± 8.45
Trp	μg/mL	17.23 ± 4.94	15.62 ± 5.77
His	μg/mL	16.78 ± 13.64	18.35 ± 14.49
Met	μg/mL	6.24 ± 1.97	6.21 ± 1.97

### Correlations of five AAs in Hyperlipidemic and healthy subjects

**Fig. 3:**
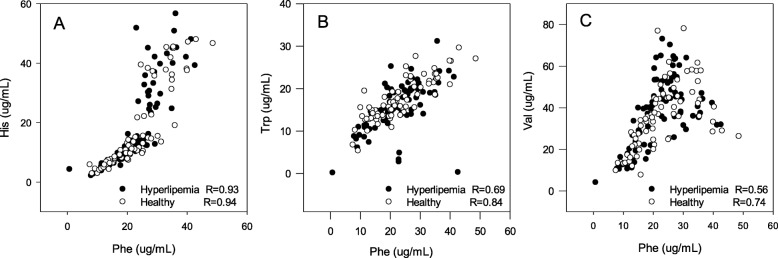
Correlation of Phe with His, Trp, and Val in 83 hyperlipidemic and 75 healthy subjects, **a**: Phe-His, **b**: Phe-Trp,**c**: Phe-Val, R: Correlation Coefficient of Spearman’s analysis

The Spearman’s correlations analysis showed that there was a high correlation among Val, Phe, Trp, His, Met in healthy and hyperlipidemic subjects (Table 5). Among them, the correlation coefficient of Phe-His was the highest, followed Phe-Trp, and Phe-Val. The scatter plots of Phe-His, Phe-Trp, and Phe-Val are showed in Fig. 3.

**Table 5:** Spearman’s correlations analysis of 5 AAs in hyperlipidemic and healthy subjects

AA	healthy subjects		hyperlipemia subjects	
	Coefficient	P	Coefficient	P
His-Phe	0.94	< 0.001	0.93	< 0.001
His-Trp	0.75	< 0.001	0.68	< 0.001
His-Met	0.40	< 0.001	0.46	< 0.001
His-Val	0.73	< 0.001	0.56	< 0.001
Phe-Trp	0.84	< 0.001	0.69	< 0.001
Phe-Met	0.51	< 0.001	0.51	< 0.001
Phe-Val	0.72	< 0.001	0.56	< 0.001
Trp-Met	0.43	< 0.001	0.44	< 0.001
Trp-Val	0.55	< 0.001	0.53	< 0.001
Met-Val	0.59	< 0.001	0.66	< 0.001

**Fig. 4:**
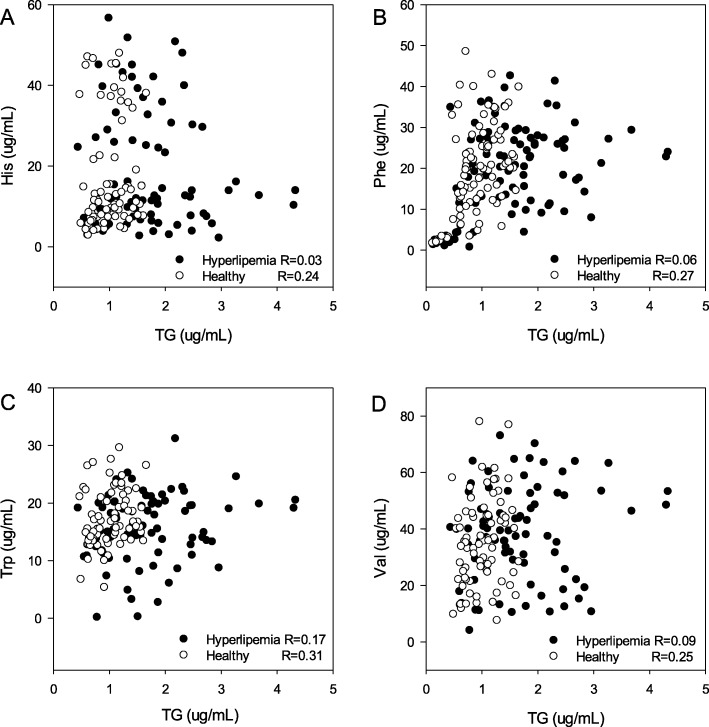
Correlation of TG with His, Phe, Trp, and Val in 83 hyperlipidemic and 75 healthy subjects, **a**: TG-His, **b**: TG-Phe, **c**: TG-Trp, **d**: TG-Val; R: Correlation Coefficient of Spearman’s analysis

The further correlations analysis revealed that the plasma level of TG was related to His, Phe, Trp, and Val in healthy subjects. The correlation coefficients of TG-His, TG-Phe, TG-Trp, and TG-Val were 0.24, 0.27, 0.31, and 0.25. Nevertheless, they decreased to 0.03, 0.06, 0.17, 0.09 in hyperlipidemic subjects (Fig. 4).

## Discussions

Commonly, hydrophilic interaction chromatography (HILIC) column is widely used to analyze amino acids [18, 23, 26–28]. HILIC column has hydrophilic polar stationary phase which can interact with highly polar compounds by strong hydrophilic interaction [18]. Being different from the HILIC column, C18 column has chains of 18 carbons attached to the silica beads, which has nonpolar column’s environment. C18 column is the most commonly used column in analytical practice. In this study, under the same conditions of UPLC-MS/MS, we found that the C18 column had better sensitivity than the HILIC column in detection of His, Met, Trp, Val, and Phe (supplement Table 5). Therefore, an ACQUITY UPLC BEH C18 Column was selected. On this column, five AAs were separated well, and no interfering endogenous substances appeared in UPLC-MS/MS chromatograms. Among 5 AAs, Val, His, Phe, and Trp have one major daughter ion, while Met has three daughter ions that is 104.02, 132.92, and 55.96 in the positive ion mode. When collision energy increased, the ion 55.96 will be the major daughter ion. Compared with other ions, the MS response of 104.02 was highest and lower background noise level. Therefore, ion 104.02 was used in this study. Based on these conditions, 5 AAs had good linearity, high accuracy, and the RSD of intra-day and inter-day precision were < 15%.

The developed UPLC-MS/MS method was successfully applied to determine the plasma of hyperlipidemic and healthy subjects. Hyperlipemia is a metabolic disease, and whether the AAs metabolism would be influenced in hyperlipemia is poorly studied. Theoretically, it is difficult for essential AAs to maintain dynamic balance when lipid metabolism is disordered. Although the concentration of AAs in plasma can be dynamically maintained in a balance by the release of endogenous proteins and the utilization of tissues [29, 30], it is different for essential AAs because they cannot be synthesized in the body. Our results indicated that the mean blood levels of Val, Phe, Trp, His, and Met were changed in hyperlipidemic subjects. The blood levels of Val and His increased and the blood level of Trp decreased.

The Spearman’s correlations analysis showed that the correlation coefficient of Phe-His was the highest, whether in the healthy subjects (0.94) or hyperlipidemic subjects (0.93). Moreover, Phe had high correlations with Trp (0.84) and Val (0.72) in healthy subjects. However, the correlation coefficients were decreased to 0.69 and 0.56 in hyperlipidemic subjects, which indicated that the level of Phe was more easily to be influenced than the other four AAs in dyslipidemia. Although the independent samples T test showed that there were no statistic differences for those 5AAs between healthy subjects and hyperlipidemic subjects. The correlation coefficients of AAs decreased in hyperlipemia, such as Phe-Trp (0.84/0.69), Phe-Val (0.72/0.56), His-Trp (0.75/0.68), and His-Val (0.73/0.56), which indicated that the balance of His, Phe, Trp, and Val had been changed. Therefore, hyperlipemia or dyslipidemia may influence the metabolic balance of His, Phe, Trp, and Val.

## Conclusions

A convenient, sensitive, and specified UPLC-MS/MS method was developed and validated for the measurement of the five essential AAs in human plasma: His, Phe, Trp, Met and Val, which can be determined within 3.0 min after precipitated by acetonitrile. For hyperlipidemic and healthy people, the level of Val was the highest, then was Phe, Trp, His and Met. The results of 5 AAs were significantly correlated. For healthy subjects, the plasma level of TG is related to that of His, Phe, Trp and Val. However, in hyperlipemia or dyslipidemia patients, a perturbation was uncovered in the metabolic balance of His, Phe, Trp and Val.

## Supplementary information

**Additional file 1 Supplement Table 1** The equation of calibration curves of 5 AAs in human plasma

**Additional file 2 Supplement Table 2** The extraction recovery, matrix effect and stability of 5 AA in human plasma (*n* = 3)

**Additional file 3 Supplement Table 3** Parameters of UPLC-MS/MS method interchanged within the range of 1–10%

**Additional file 4 Supplement Table 4** RSD values of ruggedness determined on two different columns and by different analysts

**Additional file 5 Supplement Table 5** Peaks area of 5 AAs (10 μg/mL) in pure standard and blood sample determined by Hilic and BEH C18 column

## Data Availability

All Data is available.

## Abbreviations

AAs: Amino acids
BRT: Blood routine test
GC-MS: Gas chromatography with mass spectrometry
His: Histidine
HPLC: High performance liquid chromatography
IS: Internal standard
LC-MS: Liquid chromatography tandem with mass spectrometry detection
Met: Methionine
MRM: Multiple reaction monitoring
Phe: Phenylalanine
QC: Quality-control
RE: Relative error
RSD: Relative standard deviation
TC: Total cholesterol or
TG: Triglyceride
Trp: Tryptophan
UPLC-MS/MS: Ultra-high performance liquid chromatography-tandem mass spectrometric
Val: Valine
