# Supplementary material for: Simultaneous determination of five essential amino acids in plasma of Hyperlipidemic subjects by UPLC-MS/MS
Source: Lipids Health Dis. 2020 Mar 23;19:52. doi: 10.1186/s12944-020-01216-8 (PMC7087371; doi:10.1186/s12944-020-01216-8)
Supplement: Supplementary file 1 — Additional file 1 Supplement Table 1 The equation of calibration curves of 5 AAs in human plasma [file 12944_2020_1216_MOESM1_ESM.docx]

Supplement Table 1 The equation of calibration curves of 5 AAs in human plasma

| Compound | Standard curves | Ranges (μg/mL) | R |
| --- | --- | --- | --- |
| Val | y = 0.0747x + 0.4209 | 1-100 | 0.9985 |
| Met | y = 0.1295x + 0.0605 | 1-100 | 0.9984 |
| His | y = 0.5707x + 0.3484 | 1-100 | 0.9954 |
| Phe | y = 3.9892x + 3.5217 | 1-100 | 0.9942 |
| Trp | y = 3.366x - 1.7798 | 1-100 | 0.9980 |
