# Supplementary material for: Simultaneous determination of five essential amino acids in plasma of Hyperlipidemic subjects by UPLC-MS/MS
Source: Lipids Health Dis. 2020 Mar 23;19:52. doi: 10.1186/s12944-020-01216-8 (PMC7087371; doi:10.1186/s12944-020-01216-8)
Supplement: Supplementary file 2 — Additional file 2 Supplement Table 2 The extraction recovery, matrix effect and stability of 5 AA in human plasma (n = 3) [file 12944_2020_1216_MOESM2_ESM.docx]

Supplement Table 2 The extraction recovery, matrix effect and stability of 5 AA in human plasma (n = 3)

| AA | QC  (μg/mL) | Extract recovery | | Matrix effect | | Extract stability (24h) | | |
| --- | --- | --- | --- | --- | --- | --- | --- | --- |
|  |  | Mean±SD  (%) | RSD  (%) | Mean±SD  (%) | RSD  (%) | Mean±SD  (%) | RSD  (%) |  |
| His | 10 | 74.20±9.89 | 13.33 | 70.56±5.84 | 8.27 | 93.23±3.04 | 14.27 |  |
|  | 40 | 73.39±6.19 | 8.44 | 76.65±2.71 | 3.54 | 98.08±0.50 | 10.60 |  |
|  | 80 | 81.86±4.55 | 5.56 | 78.65±6.56 | 8.33 | 97.35±1.45 | 11.31 |  |
| Met | 10 | 66.42±4.73 | 7.12 | 70.31±7.39 | 10.51 | 89.18±6.10 | 13.96 |  |
|  | 40 | 72.63±5.35 | 7.37 | 71.54±3.68 | 5.15 | 90.07±4.60 | 13.13 |  |
|  | 80 | 71.25±6.56 | 9.21 | 72.05±8.74 | 12.13 | 95.57±2.03 | 12.55 |  |
| Val | 10 | 61.80±7.27 | 11.77 | 63.72±2.75 | 4.32 | 108.59±2.90 | 14.46 |  |
|  | 40 | 68.59±3.72 | 5.42 | 77.44±3.40 | 4.39 | 94.39±2.58 | 14.88 |  |
|  | 80 | 82.50±8.41 | 10.19 | 83.97±2.83 | 3.36 | 90.03±1.18 | 11.76 |  |
| Trp | 10 | 81.07±8.64 | 10.66 | 84.14±6.76 | 8.03 | 107.77±3.81 | 5.50 |  |
|  | 40 | 65.70±8.63 | 13.13 | 67.94±2.43 | 3.58 | 107.73±3.61 | 5.64 |  |
|  | 80 | 73.42±10.03 | 13.66 | 74.10±4.78 | 6.44 | 106.72±0.26 | 5.94 |  |
| Phe | 10 | 82.62±1.56 | 1.89 | 80.46±1.79 | 2.23 | 93.46±1.41 | 10.58 |  |
|  | 40 | 62.65±8.04 | 12.83 | 62.15±1.83 | 2.95 | 95.85±4.19 | 14.67 |  |
|  | 80 | 72.21±10.20 | 14.12 | 73.93±2.26 | 3.06 | 97.55±0.65 | 6.63 |  |
