# Supplementary material for: Simultaneous determination of five essential amino acids in plasma of Hyperlipidemic subjects by UPLC-MS/MS
Source: Lipids Health Dis. 2020 Mar 23;19:52. doi: 10.1186/s12944-020-01216-8 (PMC7087371; doi:10.1186/s12944-020-01216-8)
Supplement: Supplementary file 3 — Additional file 3 Supplement Table 3 Parameters of UPLC-MS/MS method interchanged within the range of 1–10% [file 12944_2020_1216_MOESM3_ESM.docx]

Supplement Table 3 Parameters of UPLC-MS/MS method interchanged within the range of 1-10%

| AAs | QC (μg/mL) | mobile phase | pH | column temperature | flow rate |
| --- | --- | --- | --- | --- | --- |
|  | 10 | 10.48 | 8.11 | 8.89 | 8.51 |
| try | 40 | 11.98 | 13.11 | 11.16 | 5.82 |
|  | 80 | 13.70 | 11.82 | 9.39 | 1.92 |
|  | 10 | 8.18 | 8.66 | 2.82 | 3.22 |
| phe | 40 | 7.81 | 14.97 | 7.50 | 3.17 |
|  | 80 | 11.44 | 0.84 | 7.77 | 7.46 |
|  | 10 | 3.08 | 2.14 | 5.59 | 3.37 |
| his | 40 | 1.36 | 6.20 | 2.18 | 0.41 |
|  | 80 | 3.07 | 3.89 | 1.52 | 2.58 |
|  | 10 | 6.58 | 2.80 | 1.28 | 0.55 |
| met | 40 | 1.87 | 9.05 | 1.23 | 2.19 |
|  | 80 | 5.88 | 7.18 | 4.48 | 4.48 |
|  | 10 | 7.74 | 3.92 | 5.67 | 6.21 |
| val | 40 | 2.70 | 14.55 | 5.59 | 2.92 |
|  | 80 | 8.44 | 12.44 | 7.14 | 4.30 |
