# Supplementary material for: Simultaneous determination of five essential amino acids in plasma of Hyperlipidemic subjects by UPLC-MS/MS
Source: Lipids Health Dis. 2020 Mar 23;19:52. doi: 10.1186/s12944-020-01216-8 (PMC7087371; doi:10.1186/s12944-020-01216-8)
Supplement: Supplementary file 4 — Additional file 4 Supplement Table 4 RSD values of ruggedness determined on two different columns and by different analysts [file 12944_2020_1216_MOESM4_ESM.docx]

Supplement Table 4 RSD values of ruggedness determined on two different columns and by different analysts

| AAs | QC (μg/mL) | different columns | different analysts | different analysts |
| --- | --- | --- | --- | --- |
|  | 10 | 2.97 | 3.63 | 3.41 |
| try | 40 | 4.12 | 6.17 | 2.36 |
|  | 80 | 6.43 | 3.70 | 1.83 |
|  | 10 | 1.69 | 1.21 | 2.34 |
| phe | 40 | 3.35 | 1.76 | 2.08 |
|  | 80 | 1.30 | 1.24 | 2.71 |
|  | 10 | 1.44 | 5.83 | 1.43 |
| his | 40 | 2.02 | 3.53 | 4.77 |
|  | 80 | 0.96 | 1.92 | 2.32 |
|  | 10 | 14.88 | 12.94 | 13.20 |
| met | 40 | 6.87 | 8.60 | 12.87 |
|  | 80 | 11.69 | 10.95 | 7.89 |
|  | 10 | 6.85 | 1.02 | 9.15 |
| val | 40 | 12.83 | 6.90 | 5.48 |
|  | 80 | 0.53 | 6.51 | 14.24 |
