# Supplementary material for: Simultaneous determination of five essential amino acids in plasma of Hyperlipidemic subjects by UPLC-MS/MS
Source: Lipids Health Dis. 2020 Mar 23;19:52. doi: 10.1186/s12944-020-01216-8 (PMC7087371; doi:10.1186/s12944-020-01216-8)
Supplement: Supplementary file 5 — Additional file 5 Supplement Table 5 Peaks area of 5 AAs (10 μg/mL) in pure standard and blood sample determined by Hilic and BEH C18 column [file 12944_2020_1216_MOESM5_ESM.docx]

Supplement Table 5 Peaks area of 5 AAs (10μg/mL) in pure standard and blood sample determined by Hilic and BEH C18 column

| AAs | sample type | Hilic Area | BEH C18 Area | BEH/Hilic  (%) |
| --- | --- | --- | --- | --- |
|  |  | mean±SD | mean±SD |  |
| His | pure standard | 5659272.00±134790.00 | 10094845.00±219905.00 | 178.38 |
|  | blood | 127994.00±14737.52 | 1007118.75±28964.24 | 786.85 |
| Met | pure standard | 197694.50±772.50 | 255977.00±7315.00 | 129.48 |
|  | blood | 33554.50±2828.82 | 109397.67±11906.56 | 326.03 |
| Val | pure standard | 154478.50±6785.50 | 195677.50±10713.50 | 126.67 |
|  | blood | 78121.33±9510.75 | 119378.00±13201.72 | 152.81 |
| Try | pure standard | 594841.50±35814.50 | 608198.00±44869.00 | 102.25 |
|  | blood | 145602.00±16738.09 | 506416.25±17164.52 | 347.81 |
| Phy | pure standard | 3590484.50±81722.50 | 4214865.50±241810.50 | 117.39 |
|  | blood | 2713017.75±170808.20 | 4454079.00±172877.22 | 164.17 |
